# Inhibiting SLC26A4 reverses cardiac hypertrophy in H9C2 cells and in rats

**DOI:** 10.7717/peerj.8253

**Published:** 2020-01-21

**Authors:** Liqun Tang, Xiaoqin Yu, Yangyang Zheng, Ning Zhou

**Affiliations:** 1 Department of Geriatrics, Zhejiang Province People’s Hospital, Hangzhou Medical College, Hangzhou, Zhejiang, China; 2 Department of Geriatrics, Zhejiang Aid Hospital, Hangzhou, Zhejiang, China

**Keywords:** SLC26A4, Cardiac hypertrophy, Apoptosis, Autophagy, Cardiomyocytes

## Abstract

**Background:**

It has been confirmed that mutations in solute carrier family 26 member 4 (SLC26A4) contribute to pendred syndrome. However, the role of SLC26A4 in cardiac hypertrophy and the signaling pathways remain unclear.

**Methods:**

Cardiomyocytes were treated by 200 µM phenylephrine (PE) to induce cardiac hypertrophy. Also, the expression of SLC26A4, GSK3, cardiac hypertrophy markers including atrial natriuretic peptide (ANP) and brain natriuretic peptide (BNP) was detected through real-time quantitative polymerase chain reaction (RT-qPCR). Flow cytometry assay was used to test the apoptosis of PE-induced cardiomyocytes transfected by small interfere RNA (siRNA)-SLC26A4. Furthermore, we detected the expression of autophagy-related markers including light chain 3 (LC3) and P62. Finally, we established a rat model of abdominal aortic constriction (AAC)-induced cardiac hypertrophy *in vivo*.

**Results:**

RT-qPCR results showed that the mRNA expression of SLC26A4 was significantly up-regulated in PE-induced cardiac hypertrophy. After inhibiting SLC26A4, the release of ANP and BNP was significantly decreased and GSK3β was elevated *in vivo* and *in vitro*. Furthermore, inhibiting SLC26A4 promoted apoptosis of cardiac hypertrophy cells. In addition, LC3 was down-regulated and P62 was enhanced after transfection of siRNA-SLC26A4.

**Conclusion:**

Our findings revealed that SLC26A4 increases cardiac hypertrophy, and inhibiting SLC26A4 could decrease the release of ANP/BNP and promote the expression of GSK-3β *in vitro* and *in vivo*. Moreover, SLC26A4 silencing inhibits autophagy of cardiomyocytes and induces apoptosis of cardiomyocytes. Therefore, SLC26A4 possesses potential value to be a therapeutic target of cardiac hypertrophy, and our study provides new insights into the mechanisms of cardiac hypertrophy.

## Introduction

As one of the predictors of cardiovascular disease, cardiac hypertrophy is an adaptive change in cardiac overload and injury. Increased cardiac quality, protein synthesis and re-expression of fetal-type genes are major characteristics of cardiac hypertrophy (Xue et al., 2017a). However, persistent cardiac hypertrophy contributes to heart dysfunction and ultimately heart failure (Liu et al., 2018a). Also, heart failure is considered to be the leading cause of death in the elderly (Liu, Liu & Wang, 2018). Therefore, reducing cardiac hypertrophy is an effective clinical treatment strategy against heart failure (Xue et al., 2017b). Pathological cardiac hypertrophy is a complex disease process that results from primary heart disease such as hypertension, valvular heart disease and coronary artery disease (Guan et al., 2017; Chu et al., 2018). In addition, there is a lack of effective methods to prevent cardiac hypertrophy. Therefore, it is necessary to identify potential targets or pathways that may modulate cardiac hypertrophy.

As reported before, myocardial fibrosis is a major feature of pathologic cardiac hypertrophy, leading to ventricular dysfunction and arrhythmias (Zhao et al., 2017). Presently, α smooth muscle actin (α-SMA) is the hallmark of mature myofibroblasts (Zhang, Zhang & Wang, 2018). GSK-3β is a ubiquitously expressed constitutively active serine/threonine kinase that phosphorylates cellular substrates and regulates a variety of cellular functions (Hardt & Sadoshima, 2002). There is growing evidence that GSK-3β is an important regulator of cardiac hypertrophy (Hardt & Sadoshima, 2004). Therefore, GSK-3β plays an important role in regulating heart development.

Apoptosis is programmed cell death which normally ensures proper functional and metabolic homeostasis in multicellular organisms (Wang et al., 2016). Autophagy is an evolutionarily conservative process (Qi et al., 2019). Central to this process is the formation of autophagosomes, which is controlled by a set of autophagy related genes (Atgs), such as Beclin-1, LC3, and P62  (Li, Wang & Yang, 2015). Beclin1 and LC3 play a key role in the formation of autophagosomes (Gu et al., 2016). During the formation of autophagosomes, cytosolic LC3-I is conjugated to phosphatidylethanolamine to generate LC3-II, which is recruited to the autophagosomal membrane and degraded followed by the fusion of autophagosomes to lysosomes, thus, the LC3-II/LC3-I ratio is a marker of autophagy activity. It has been found that changes in autophagosome-lysosomal pathway activity have key pathogenic effects in cardiac hypertrophy (Ba et al., 2019). Autophagy at the normal levels protects cardiomyocytes from environmental stimuli, but autophagy imbalance can lead to cardiac hypertrophy (Xie et al., 2018). Therefore, cardiomyocyte autophagy plays an important role in maintaining myocardial function. Although much progress in the identification of genes and signaling pathways involved in this process, additional regulatory mechanisms still remain to be clarified. Thus, exploring novel molecular mechanisms mediating cardiac hypertrophy need to be addressed.

SLC26A4, also known as the PDS gene, has 21 exons encoding a multi-transmembrane protein Pendrin containing 780 amino acids (Nonose et al., 2018; Belguith-Maalej et al., 2011). Growing studies found that SLC26A4 plays a critical role in various diseases such as thyroid cancer and gastric cancer (Bastos et al., 2015; Xing et al., 2003; Zane et al., 2013; Li et al., 2016). More importantly, recent research found that SLC26A4 is associated with hypertension and left ventricular hypertrophy index is significantly reduced in patients with SLC26A4 mutation, indicating that SLC26A4-AS1 might participate in the processes of cardiac hypertrophy (Kim et al., 2017). However, the role of SLC26A4 in cardiac hypertrophy and relevant signaling pathways remain unclear. Therefore, in our study, we explored the role of SLC26A4 in cardiac hypertrophy, and whether inhibiting SLC26A4 would markedly improve cardiac hypertrophy.

## Materials and Methods

### Cell culture and treatments

H9C2 cells (an embryonic rat myocardium-derived cell line) were obtained from the Cell Bank of Sai Lan Biological Technology Co., Ltd. (Zhejiang, China). H9C2 cells were cultured in DMEM medium (SH30243.01B; Hyclone, USA) containing 10% foetal bovine serum (FBS; SH30084.03; Hyclone) 10% L-glutamine, 0.5% penicillin/streptomycin, 10% nonessential amino acids and 10% pyruvate at 37 °C, 5% CO_2_ and saturated humidity. The media was refreshed every 3 days. H9C2 cells cultured to approximately 80% confluence were treated by PE (200 µM) for 48 h to induce cardiac hypertrophy.

### Immunofluorescence staining and confocal microscopic assay

Immunofluorescence staining was performed to detect the expression of α-SMA in myocardial cells. Briefly, the cells were cultured on the coverslips. Next, the cell suspension was transferred to a culture plate and incubated at 37 °C in a 5% CO_2_ water bath incubator for 2–3 days. When the adherent cells were grown to cover 2/3 of the bottom of the culture plate, the plate was taken out to obtain a slide of cells. Then the cells were fixed with 4% paraformaldehyde (Sangon Biotech Co., Ltd., Shanghai, China) for 15 min, and permeabilized using 0.5% Triton X-100 (sigma, USA) for 20 min at room temperature. Next, the cells were blocked and incubated with primary antibody anti-α-SMA antibody (Proteintech Group, Inc., Wuhan, China) at 4 °C overnight. After that, the cells were incubated with secondary antibody (Proteintech Group, Inc., Wuhan, China) in the dark. The DAPI (sigma, USA) was counterstained for 5 min in the dark in order to identifying nucleus. Finally, photographs was observed under a confocal fluorescence microscope (Olympus Corporation, Japan).

### RNA extraction and RT-qPCR

Total RNA was extracted from cells or tissues using Trizol reagent (Invitrogen, Carlsbad, CA). The RNA purity and concentration were determined according to the ratio 260/280 nm in a UV spectrophotometer. Total RNA was reverse transcribed into cDNA using the RevertAid First Strand cDNA synthesis Kit (Thermo). qPCR detection was subsequently performed using SsoAdvance Universal SYBR Green Supermix (BIO-RAD). The experimental results were automatically calculated by the quantitative PCR analysis software BIO-RAD CFX Manager 3.1. The primers of target genes were as follows: rat SLC26A4, 5′-CATCATGCCTGGCTGGTTCT-3′ (F), 5′-TGGACACCAACATTCCGTCA-3′ (R); rat Glyceraldehyde-3-phosphate dehydrogenase (GAPDH), 5′-GGAGCGAGATCCCTCCAAAAT-3′ (F), 5′-GGCTGTTGTCATACTTCT CATGG-3′ (R). GAPDH served as an internal control. The relative expression level of target genes was determined using the 2^−ΔΔ*Ct*^ method.

### Cell transfection

SLC26A4 siRNA or the control siRNA was inserted into the pcDNA3.1 vector (TAKARA, Beijing, China). H9C2 cells were transfected with the plasmids via lipofectamine reagent (Invitrogen, USA) for 24 h. The sequence of siRNA-SLC26A4 was as follows: 5′-GCUGCAGUUGCUCAAGAAATT-3′ (F), 5′-UUUCUUGAGCAACUGCAGCTT-3′ (R).

### Western blot

Total proteins were extracted from cells using the RIPA lysis buffer (P0013B; Beyotime Biotechnology, Shanghai, China), and then then centrifuged at 4 °C 12,000 g for 15 min. The supernatant was harvested, and protein concentrations were detected by BCA protein assay (P0009; Beyotime, Shanghai, China). Then, the extracted proteins were separated by SDS-PAGE, which were transferred to a PVDF membrane. After that, the membrane was blocked with 5% skim milk for 1 h at room temperature. Next, the membrane was incubated with primary antibodies overnight at 4 °C, followed by incubation with horseradish peroxidase-labeled goat anti-rabbit secondary antibody (1/5,000) for 1 h at room temperature. The proteins were visualized with Enhanced Luminol Reagent and Oxidizing Reagent, and the results were detected using a gel imaging system. The primary antibodies were as follows: α-SMA (1:1,000; Sangon Biotech Co., Ltd., Shanghai, China), beclin-1 (1:1,000; Sangon Biotech Co., Ltd.), LC3 (1/1,000; Sangon Biotech Co., Ltd.), P62 (1/1,000; Sangon Biotech Co., Ltd.), GAPDH (1/5,000, Atagenix, Wuhan, China). GAPDH was used as an internal control.

### Flow Cytometry of cell apoptosis

Flow cytometry assay was used to test the apoptosis of PE-induced cardiomyocytes transfected by siRNA-SLC26A4. Cell apoptosis was analyzed using the Annexin V-FITC Apoptosis Detection Kit according to the manufacturer’s instructions. Briefly, 100  µL cell suspension was prepared. After that, 5 µl Annexin V-FITC and 10 µl propidium iodide (PI) (20  µg/ml) were added and incubated in the dark for 15 min at room temperature. The cell apoptosis was analyzed using a flow cytometry (Beckman, USA). The apoptosis rate (%) = the number of apoptotic cells/total number of cells.

### Autophagy flux assay

To evaluate the autophagy flux, H9C2 cells were transfected with green fluorescent protein with microtubule-associated protein LC3 (GFP-LC3) to monitor puncta formation and visualized under a confocal microscope. At 48 h post-adenovirus transduction, H9C2 cells were subjected to NC, PE, siRNA-SLC26A4, PE + NC and PE + siRNA-SLC26A4. The presence of autophagosomes and autolysosomes was analyzed by confocal microscopy.

### Animals and treatments

A total of ten healthy male Sprague Dawley (SD) rats weighed 250  ± 20 g were purchased from Shanghai Experimental Animal Center (Shanghai, China), which were acclimated to the laboratory environment for 1 week. Hangzhou Medical College provided full approval for this research (20190232). All rats were randomly divided into three groups (*n* = five per group): normal group, cardiac hypertrophy group, cardiac hypertrophy + siRNA-SLC26A4 group. The cardiac hypertrophy was induced by AAC. In brief, rats were anesthetized by intraperitoneal injection of 10% chloral hydrate (0.3 ml/100 g), and then an incision of about 2 cm was cut vertically under the right rib to non-invasively separate tissue fat around the right kidney. A 6-0 sterile thread was passed through the isolated abdominal aorta. According to a suitable pad (200–230 g: 23-gauge; 230–250 g: 22-gauge) on the rat body, the wire was tied and the pad was pulled out, causing about 60% stenosis. Then, the excess thread was cut, 0.5 ml of physiological saline was injected, and the muscle layer and the skin layer were sutured with a 5-0 silk thread, and iodine was disinfected. Postoperative penicillin 200,000 U/day continuous intramuscular injection was undergone for one week to prevent infection. A similar surgery was performed in the sham-operated mice with the exception of aortic banding. The siRNA-SLC26A4 powder (5OD) was dissolved with 20 ul of ddH2O. To study the effect of siRNA-SLC26A4, the mice were injected with siRNA-SLC26A4 (2.5 ul) once a day for seven days. The control mice were injected with an equal volume of normal saline. After seven days, all mice were sacrificed after an overdose of 2% sodium pentobarbital. Rat hearts were removed. Rat hearts were harvested and immediately frozen in liquid nitrogen for total RNA extraction or immersed in 4% formalin at room temperature, followed by paraffin embedding. RT-qPCR was used to detect the expression of SLC26A4, GSK-3β and hypertrophic markers including ANP and BNP. The primers of target genes are listed in Table 1. Mouse body weights were recorded before the animals were euthanized.

**Table 1:** The primers of target genes.

Target gene	5′–3′ sequence
ANP	5′-GGGCTTCTTCCTCTTCCTG-3′ (F)
	5′-CGCTTCATCGGTCTGCTC-3′ (R)
BNP	5′-AAGTCCTAGCCAGTCTCCA-3′ (F)
	5′-GGTCTATCTTCTGCCCAAA-3′ (R)
GSK-3β	5′-ACTATGTCAACTCTGGCTATGC-3′ (F)
	5′-TCGGTAAGTGCGAGGAAT-3′ (R)
GAPDH	5′-ACTCCCATTCTTCCACCTTTG-3′ (F)
	5′-CCCTGTTGCTGTAGCCATATT-3′ (R)

### Haematoxylin–Eosin (H&E) staining

The fresh rat hearts were fixed with 4% paraformaldehyde for 24 h at room temperature. After dehydration, the wax-impregnated hearts were embedded in paraffin. After the wax was solidified, the trimmed wax block was placed on a paraffin slicer and sliced to 4-µm thickness, followed by dewaxing of the paraffin section. Next, hematoxylin was used to stain the nucleus for 5–10 min. Eosin was used to stain the cytoplasm for 1–3 min. Then, the dehydrated sealing piece was placed, and the slice was dehydrated, followed by neutral gum seal. Finally, the staining results were observed by upright optical microscopy (Olympus Corporation, Japan). The sections were incubated with primary antibodies including anti-ANP (Sangon Biotech Co., Ltd.), anti-BNP (Sangon Biotech Co., Ltd.) and anti-GSK3β (Sangon Biotech Co., Ltd.) overnight, followed by secondary antibody.

### Statistical analysis

All statistical analyses were performed using Graphpad Prism software (Graph Pad Software, Inc., San Diego, CA, USA). All experiments were repeated at least three times. The data are presented as mean ± standard deviation (SD). Two group and multiple group comparisons were determined by Student’s *t*-test and one-way ANOVA, respectively (Liang et al., 2018). *P*-value <0.05 was considered statistically significant.

## Results

### SLC26A4 is up-regulated in PE-induced cardiac hypertrophy

**Figure 1:**
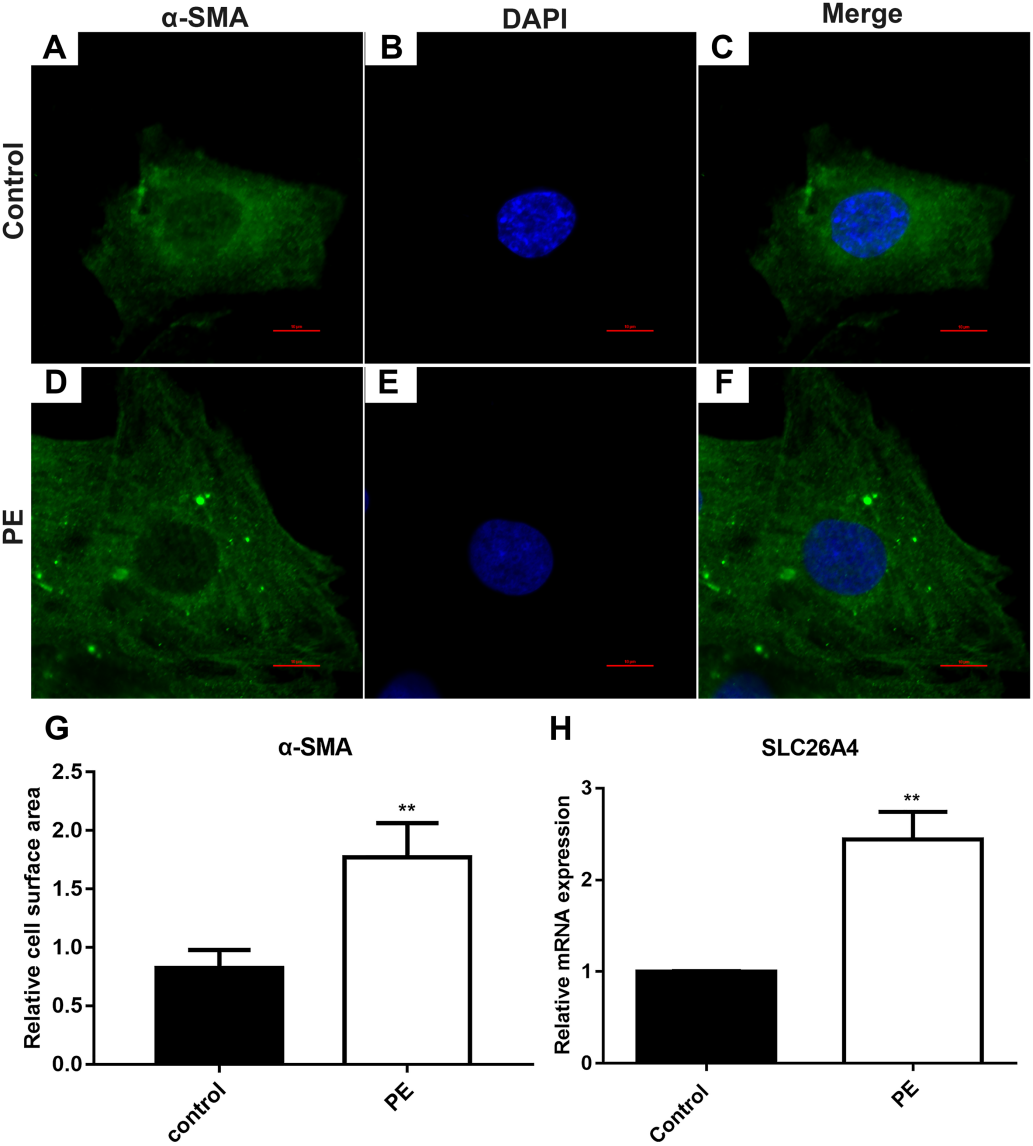
SLC26A4 is up-regulated in PE-induced cardiac hypertrophy. (A–F) Morphological changes in H9C2 cells treated with PE. Morphological changes in cardiomyocytes were examined by α-SMA immunofluorescence staining, followed by fluorescence microscopy. The nucleus was stained with DAPI (blue). Scale bar shows 10 µm. (G) The cell surface area was measured using anti-α-SMA staining (green) under fluorescence microscopy. (H) The relative mRNA expression level of SLC26A4 was detected in PE-induced cardiac hypertrophy by RT-qPCR. GAPDH served as an internal control. The relative expression level of SLC26A4 was determined using the 2^−ΔΔ*Ct*^ method. All experiments were performed at least three times. Data represent mean ± SD. **P-value < 0.01.

The cardiomyocytes were treated by PE to induce cardiac hypertrophy *in vitro*. Cardiomyocytes treated with PE is a method used to generate the features of pathological hypertrophy, like increased cell size *in vitro* (Taylor, Rovin & Parsons, 2000). Firstly, we tested whether cardiac hypertrophy was successfully induced by 200 µM PE. Morphological changes in these cardiomyocytes were observed using confocal microscopy (Figs. 1A–1F). Immunofluorescence and confocal microscopic assay results demonstrated that the expression of α-SMA in PE-induced cardiac hypertrophy was significantly higher than that in control group. The relative cell surface area was increased in H9C2 cells treated by PE in Fig. 1G. Therefore, in our study, cardiac hypertrophy was successfully induced by 200 µM PE. Then we observed the expression of SLC26A4 in H9C2 cells treated with PE. RT-qPCR results showed that the mRNA expression of SLC26A4 was significantly up-regulated in PE-induced cardiac hypertrophy in Fig. 1H.

### Inhibiting SLC26A4 ameliorates PE-induced cardiac hypertrophy

**Figure 2:**
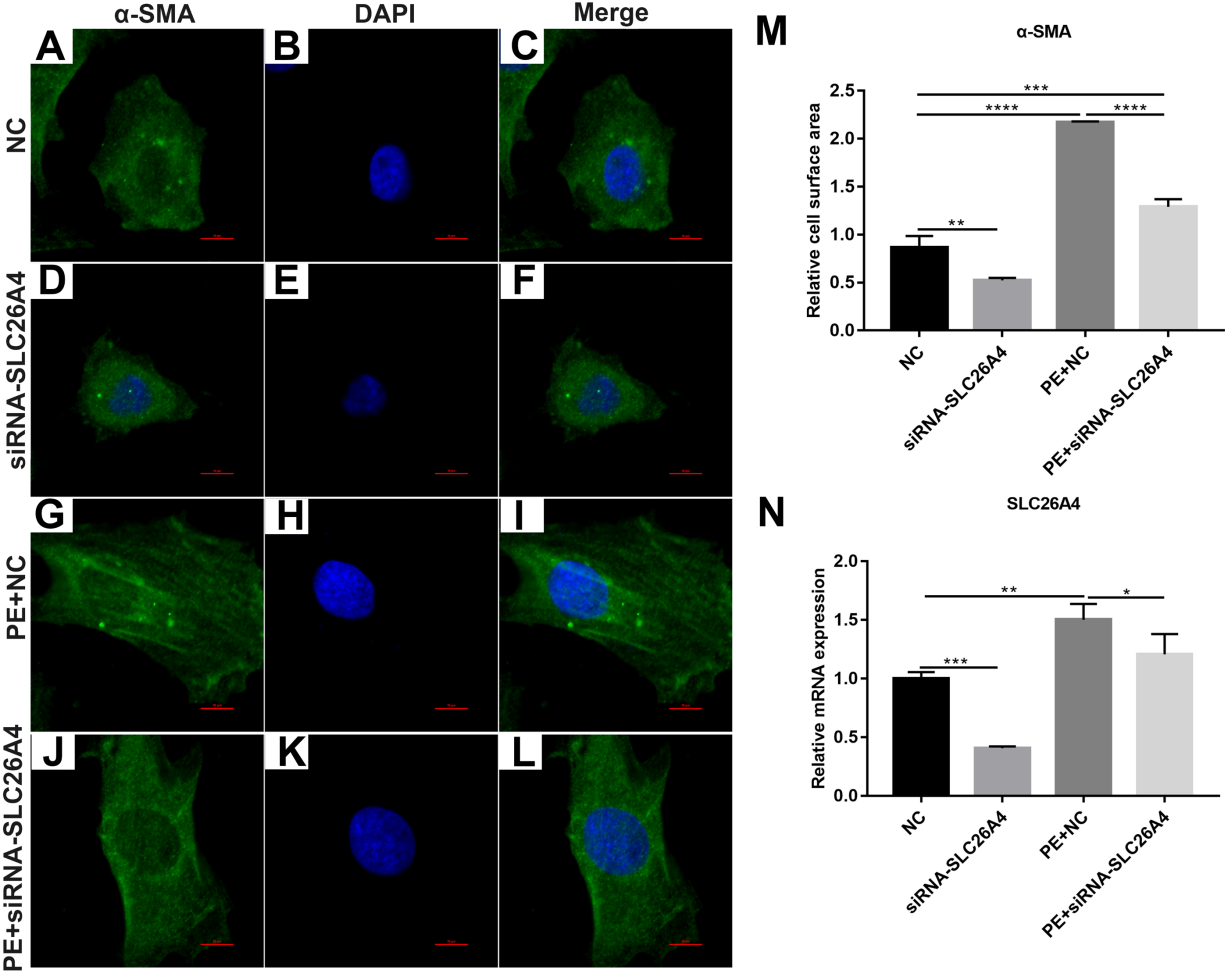
Inhibiting SLC26A4 ameliorates PE-induced cardiac hypertrophy. (A–L) Morphological changes in PE-induced H9C2 cells transfected by siRNA-SLC26A4. Morphological changes in cardiomyocytes were assessed by α-SMA immunofluorescence staining, followed by fluorescence microscopy. The nucleus was stained with DAPI (blue). Scale bar shows 10 µm. (M) The cell surface area was measured using anti-α-SMA staining (green) under fluorescence microscopy. (N) RT-qPCR results showing the relative mRNA expression level of SLC26A4 in PE-induced H9C2 cells transfected by siRNA-SLC26A4. The relative expression level of SLC26A4 was determined using the 2^−ΔΔ*Ct*^ method. All experiments were performed at least three times. Data represent mean ± SD. *P-value < 0.05; **p-value < 0.01; ***p-value < 0.001; ****p-value < 0.0001.

We found that SLC26A4 was up-regulated in PE-induced H9C2 cells, indicating that SLC26A4 could participate in the processes of cardiac hypertrophy. Therefore, we asked that whether inhibiting SLC26A4 could reverse PE-induced cardiac hypertrophy. Firstly, immunofluorescence and confocal microscopic assay results showed that the relative cell surface area in PE-induced H9C2 cells transfected by siRNA-SLC26A4 was significantly decreased compared with control group (Figs. 2A–2M). Furthermore, we tested the expression of SLC26A4 in H9C2 cells transfected by siRNA-SLC26A4. RT-qPCR results showed that SLC26A4 was significantly down-regulated in H9C2 cells transfected by siRNA-SLC26A4. Furthermore, we observed that the expression of SLC26A4 was down-regulated in PE-induced H9C2 cells transfected by siRNA-SLC26A4 compared with PE-induced H9C2 cells in Fig. 2N. Above results suggest that inhibiting SLC26A4 could ameliorate PE-induced cardiac hypertrophy.

**Figure 3:**
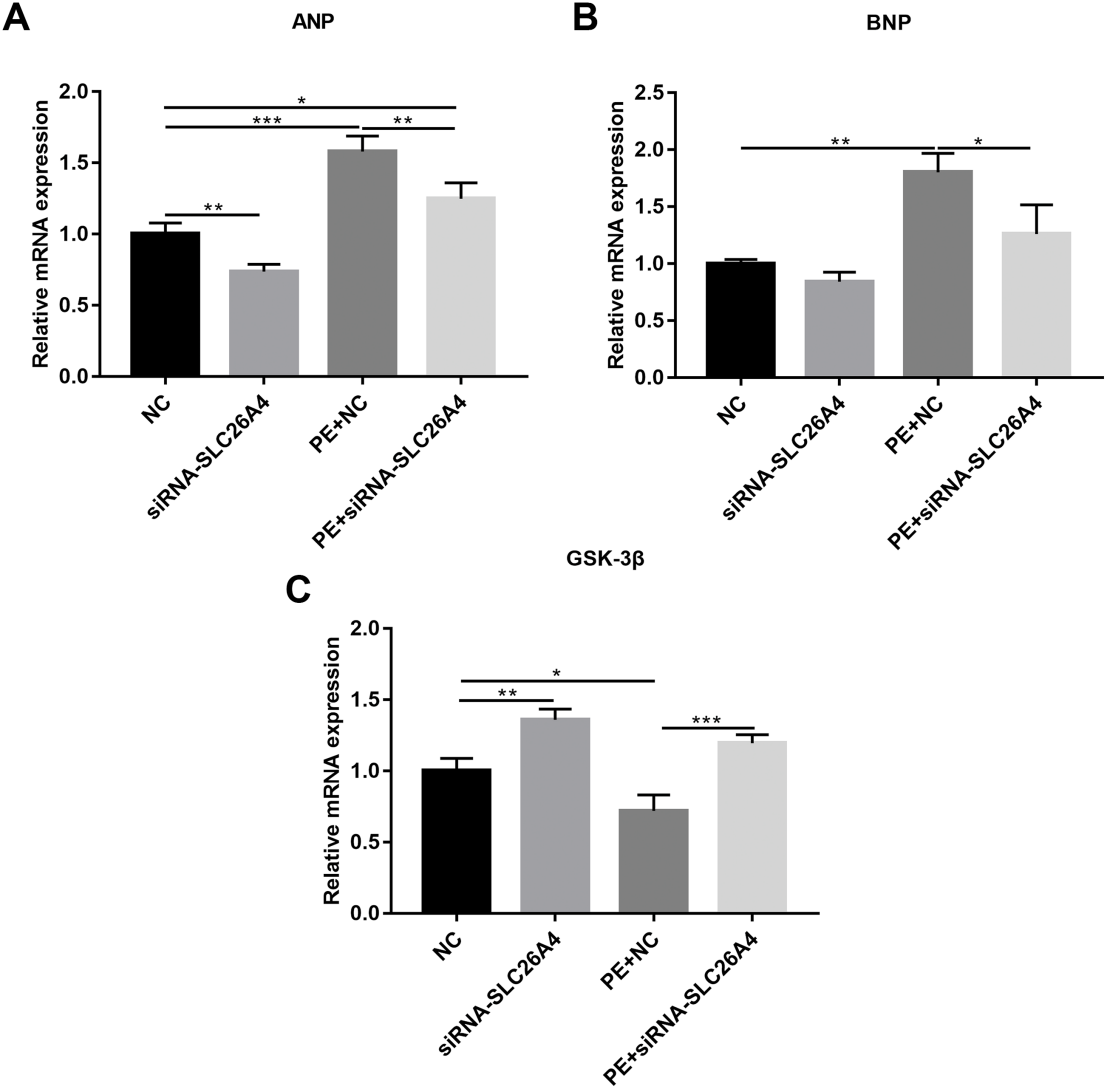
Inhibiting SLC26A4 ameliorates the expression levels of cardiomyocyte hypertrophy markers in PE-induced cardiac hypertrophy. RT-qPCR results showing the relative mRNA expression levels of (A) ANP; (B) BNP and (C) GSK3β in PE-induced H9C2 cells transfected by siRNA-SLC26A4. The relative expression level of ANP, BNP and GSK3β was determined using the 2^−ΔΔ*Ct*^ method. All experiments were performed at least three times. Data represent mean ± SD. *P-value < 0.05; **p-value < 0.01; ***p-value < 0.001.

We also observed the expression levels of cardiac hypertrophy markers including ANP and BNP. RT-qPCR results revealed that the expression levels of ANP and BNP were both elevated in PE-induced H9C2 cells. When PE-induced H9C2 cells were transfected by siRNA-SLC26A4, the expression levels of ANP and BNP were both significantly decreased in Figs. 3A and 3B. In addition, the mRNA expression level of GSK3β was also tested. The results showed that in PE-induced H9C2 cells transfected by siRNA-SLC26A4, the expression of GSK3β was significantly elevated (Fig. 3C). These results demonstrate that inhibiting SLC26A4 could ameliorate the expression levels of cardiac hypertrophy markers.

### Inhibiting SLC26A4 promotes cell apoptosis in PE-induced cardiac hypertrophy

**Figure 4:**
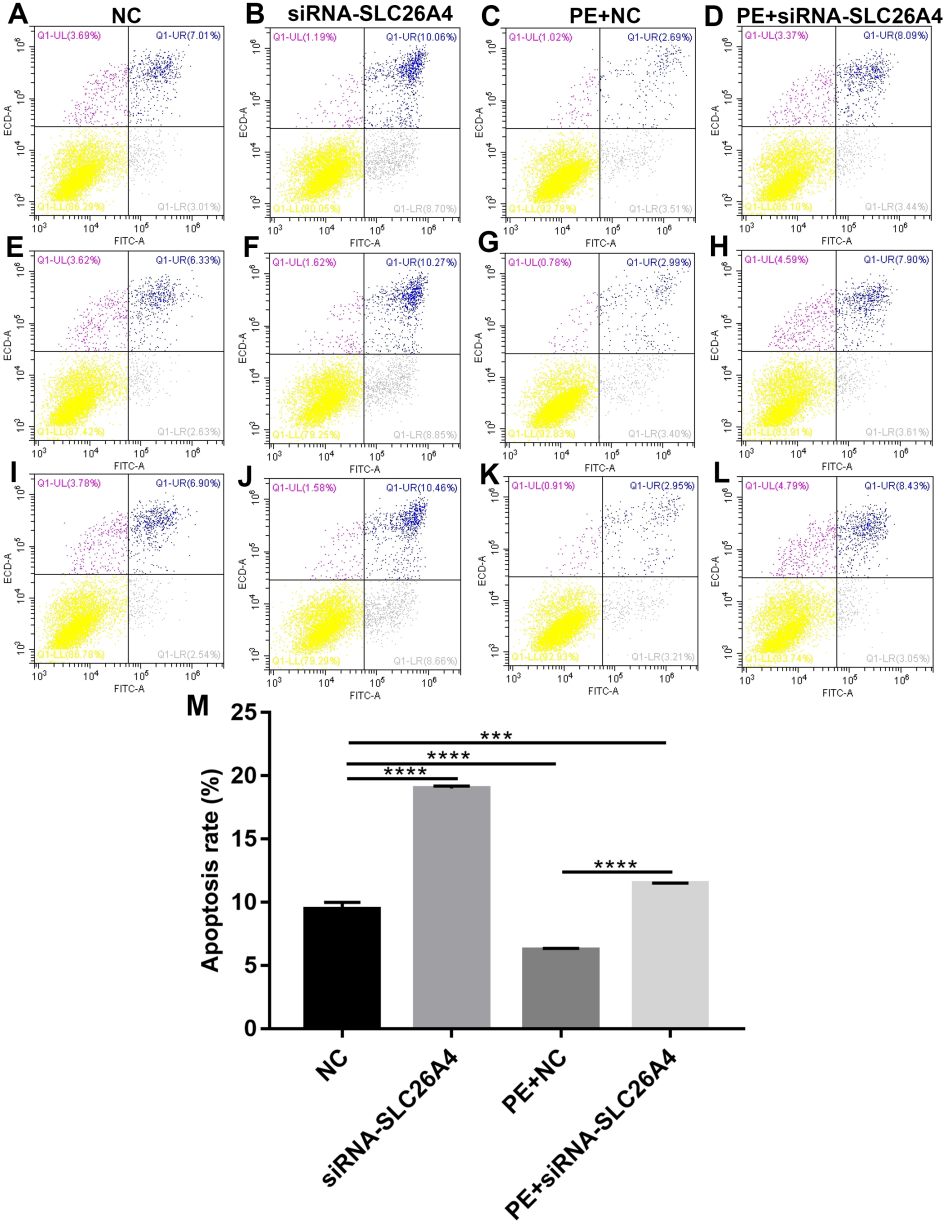
Inhibiting SLC26A4 promotes cell apoptosis in PE-induced cardiac hypertrophy. (A–L) Flow cytometry assay results showing the apoptosis of PE-induced H9C2 cells transfected by siRNA-SLC26A4. (M) The apoptosis rate of PE-induced H9C2 cells transfected by siRNA-SLC26A4. All experiments were performed at least three times. Data represent mean ± SD. **p-value < 0.01; ***p-value < 0.001; ****p-value < 0.0001.

We further investigated whether SLC26A4 could affect cell apoptosis in cardiac hypertrophy. Flow cytometry assay results showed that the apoptosis rate of H9C2 cells transfected by siRNA-SLC26A4 was increased compared with control group (Figs. 4A–4L). Moreover, compared with PE-induced H9C2 cells, the apoptosis rate was significantly elevated in PE-induced H9C2 cells transfected by siRNA-SLC26A4 in Fig. 4M. Above results reveal that inhibiting SLC26A4 promotes cell apoptosis in cardiac hypertrophy.

### Inhibiting SLC26A4 attenuates cell autophagy in PE-induced cardiac hypertrophy

**Figure 5:**
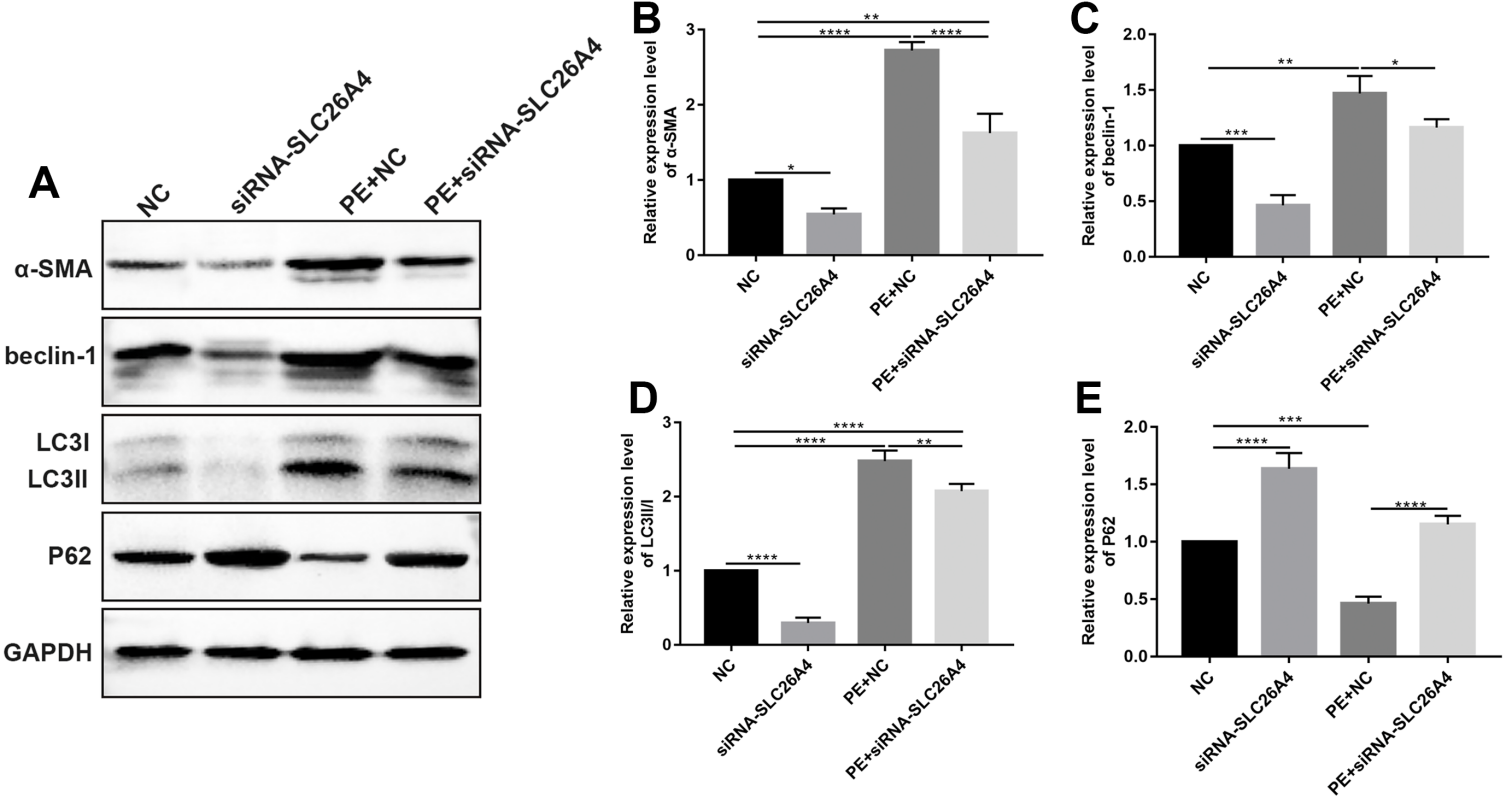
Inhibiting SLC26A4 attenuates cell autophagy in PE-induced cardiac hypertrophy. (A) Representative images of western blots. Western blot analysis results showing the expression levels of α-SMA (B) and autophagy-related proteins including beclin-1 (C), LC3II/I (D) and P62 (E) in PE-induced H9C2 cells transfected by siRNA-SLC26A4. All experiments were performed at least three times. Data represent mean ± SD. *p-value < 0.05; **p-value < 0.01; ***p-value < 0.001; ****p-value < 0.0001.

According to immunofluorescence and confocal microscopic assay results, we found that the expression of α-SMA in PE-induced H9C2 cells was significantly elevated. We further examined the expression level of α-SMA by western blot (Figs. 5A and 5B). Consistent with our previous study, western blot results showed that inhibiting SLC26A4 significantly increased the expression of α-SMA in PE-induced H9C2 cells.

**Figure 6:**
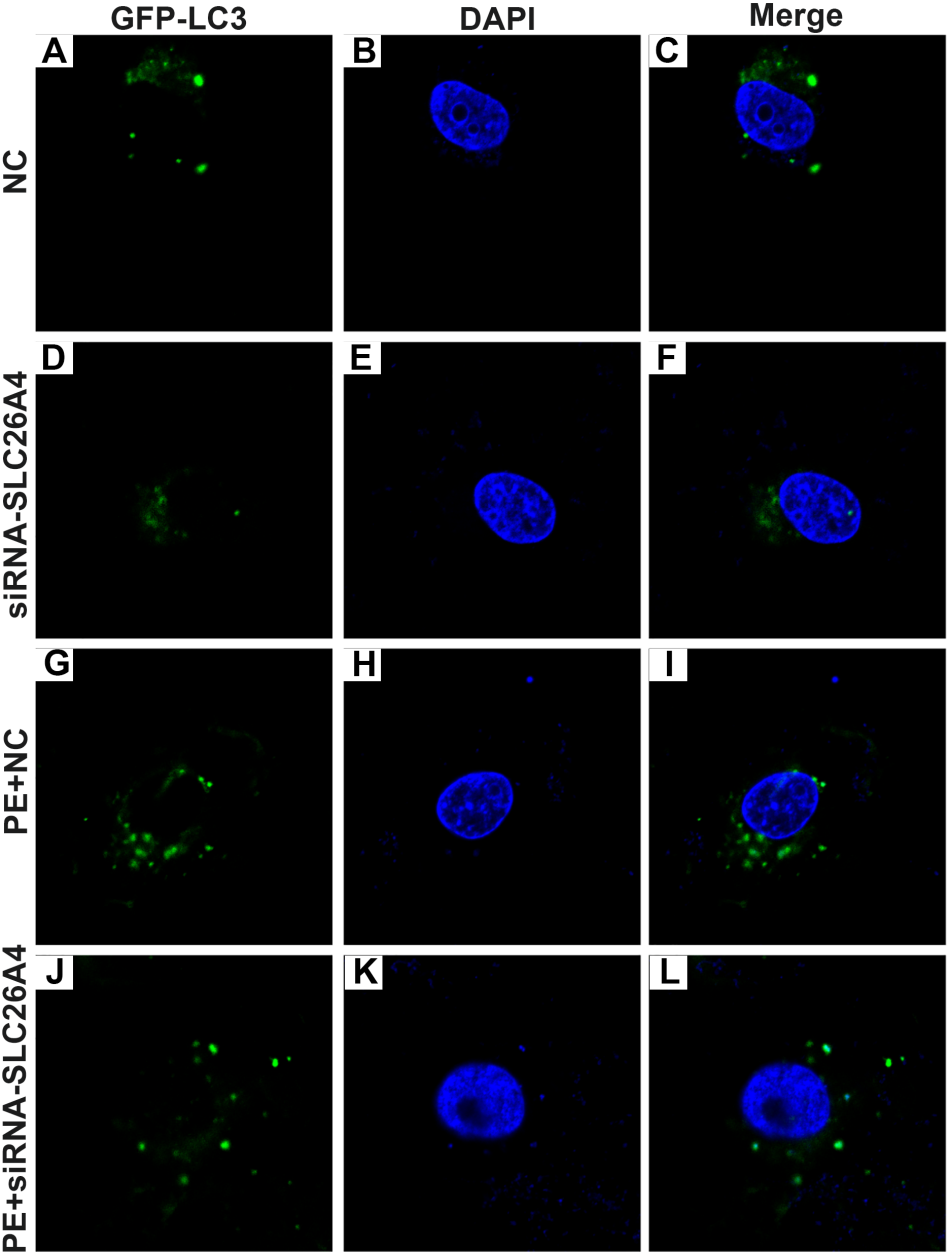
Effect of PE and siRNA-SLC26A4 on autophagy flux in H9C2 cells transfected with GFP-LC3. Microscopy showed that siRNA-SLC26A4 treatment increased the autophagy flux in PE-induced H9C2 cells (A–L).

Autophagy is in association with the pathological processes of cardiac hypertrophy. To further observe the role of SLC26A4 in cell autophagy, we examined the expression of autophagy-related markers including beclin-1, LC3II/I and P62 in PE-induced H9C2 cells transfected by SLC26A4. As shown in Figs. 5C–5E, inhibiting SLC26A4 significantly elevated the expression of beclin-1, LC3II/I and P62 in PE-induced H9C2 cells. Autophagy is a dynamic process that involves autophagosome formation and lysosome degradation. To evaluate the autophagy flux, H9C2 cells were transfected with GFP-LC3. Microscopy showed that siRNA-SLC26A4 treatment decreased the autophagy flux in PE-induced H9C2 cells, which changed to a diffuse pattern in response to PE (Figs. 6A–6L). Therefore, in PE-induced cardiac hypertrophy, inhibiting SLC26A4 could attenuate cell autophagy.

### Inhibiting SLC26A4 could reverse PE-induced cardiac hypertrophy *in vivo*

**Figure 7:**
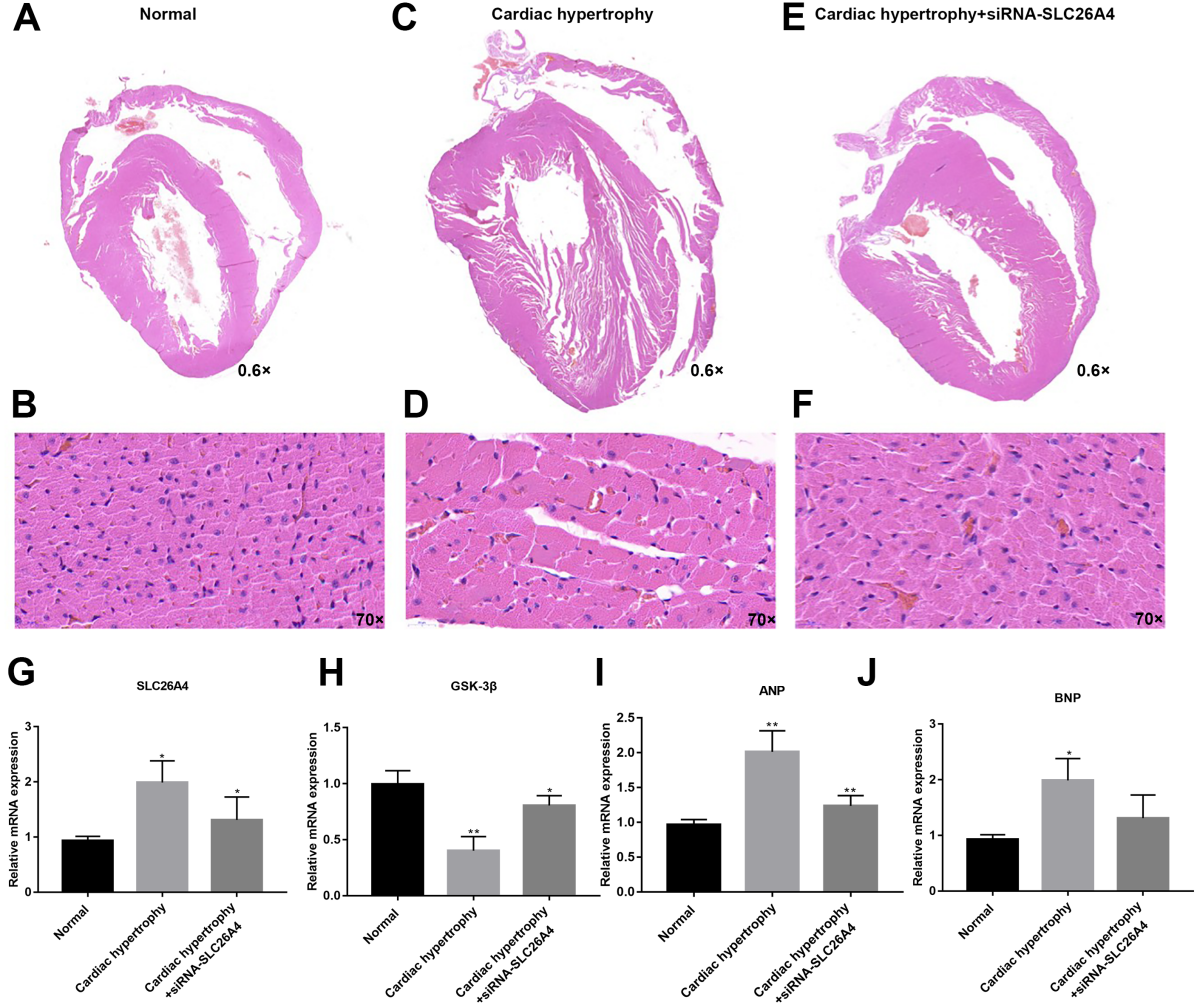
Inhibiting SLC26A4 could reverse PE-induced cardiac hypertrophy *in vivo*. (A–F) Histological sections were stained with H&E to detect cardiac hypertrophy (0.6×, 70×). RT-qPCR results showed the mRNA expression levels of SLC26A4 (G), GSK-3β (H), ANP (I) and BNP (J) in PE-induced cardiac hypertrophy injected with siRNA-SLC26A4. The relative expression levels of SLC26A4, GSK-3β, ANP and BNP was determined using the 2^−ΔΔ*Ct*^ method. All experiments were performed at least three times. Data represent mean ± SD. *p-value < 0.05; **p-value < 0.01.

**Figure 8:**
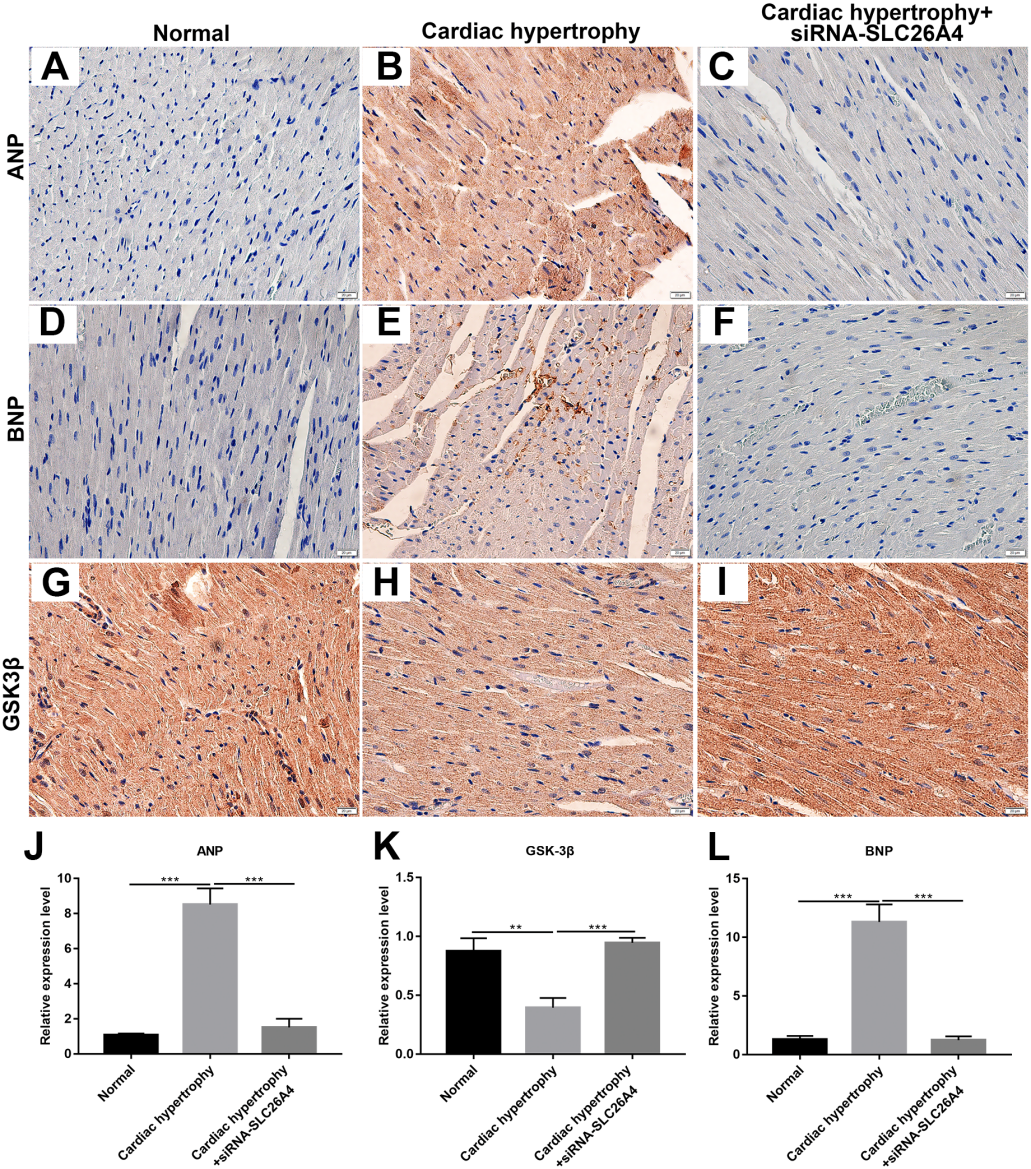
Inhibiting SLC26A4 could inhibit the expression of ANP and BNP and promote GSK-3β accumulation. (A–I) Representative images of immunohistochemistry. Immunohistochemistry results showing the expression level of ANP (J), GSK-3β (K) and BNP (L) in PE-induced cardiac hypertrophy injected with siRNA-SLC26A4. All experiments were performed at least three times. Data represent mean ± SD. **p-value < 0.01; ***p-value < 0.001.

We further confirmed whether inhibiting SLC26A4 could reverse PE-induced cardiac hypertrophy. We established PE-induced cardiac hypertrophy models *in vivo*. The siRNA-SLC26A4 was injected into PE-induced cardiac hypertrophy models. After seven days, rat hearts were taken out. H&E staining results showed that cardiac hypertrophy was markedly ameliorated after injection with siRNA-SLC26A4 (Figs. 7A–7F). In addition, RT-qPCR results demonstrated that SLC26A4 expression was inhibited in PE-induced cardiac hypertrophy mice injected with siRNA-SLC26A4 (Fig. 7G). Inhibiting SLC26A4 significantly elevated the mRNA expression of GSK-3β in PE-induced cardiac hypertrophy models (Fig. 7H). Furthermore, the expression levels of hypertrophic markers including ANP and BNP were significantly decreased in rat hearts after injection with siRNA-SLC26A4 (Figs. 7I and 7J). Consistent with RT-qPCR results, immunohistochemistry results showed that siRNA-SLC26A4 significantly attenuated the expression levels of ANP and BNP in PE-induced cardiac hypertrophy models (Figs. 8A–8L). However, siRNA-SLC26A4 significantly promoted the expression level of GSK-3β in PE-induced cardiac hypertrophy models (Figs. 8A–8L).

Therefore, inhibiting SLC26A4 could reverse PE-induced cardiac hypertrophy *in vivo*, indicating that SLC26A4 could become a potential therapeutic target for cardiac hypertrophy.

## Discussion

Cardiac hypertrophy is the compensatory response of the heart to various stresses. Persistent cardiac hypertrophy contributes to heart failure. Current research has revealed some molecular targets and regulatory factors for cardiac hypertrophy, however, its underlying molecular mechanisms have not been well elucidated (Qi et al., 2019; Wo et al., 2018). Apoptosis and autophagy play a key role in the maintenance of cardiac function. However, the role of apoptosis and autophagy in the cardiac hypertrophy remains to be determined (Li et al., 2018). In the present study, we showed that inhibiting SLC26A4 attenuates cardiac hypertrophy by apoptosis and autophagy.

In our study, cardiac hypertrophy was successfully induced by 200 µM PE *in vitro*. Myocardial fibrosis is usually accompanied by cardiac hypertrophy, which has become an important pathophysiological characteristic of the development of cardiac hypertrophy (Liu et al., 2018b). In this study, we found that the expression of myocardial fibrosis-related protein α-SMA was elevated in myocardial cells treated with PE. Furthermore, cardiac hypertrophy is characterized by an increased cell size. Therefore, we calculated the relative surface area of myocardial cells. Our results showed that the cell surface area was increased after cardiomyocytes treated by PE as seen via immunofluorescence and confocal microscopic assays. However, inhibiting SLC26A4 significantly reversed the increased α-SMA and cell surface area induced by PE, indicating that inhibiting SLC26A4 could attenuate myocardial fibrosis and cardiac hypertrophy.

In PE-induced cardiac hypertrophy, we found that SLC26A4 was significantly elevated. Intriguingly, the expression of SLC26A4 was significantly decreased in cardiomyocytes transfected with siRNA-SLC26A4 compared to controls. However, siRNA-SLC26A4 treatment only slightly attenuated its increased expression levels induced by PE in cardiomyocytes, indicating that high SLC26A4 expression could play an important role in cardiac hypertrophy. Our results showed that inhibiting SLC26A4 suppressed cardiac hypertrophy, as determined by the down-regulation of ANP and BNP (Li et al., 2017) and the up-regulation of GSK-3β. We also observed the expression of ANP, BNP and GSK-3β in cardiac hypertrophy models *in vivo*. Consistent with the results *in vitro*, siRNA-SLC26A4 inhibited ANP and BNP and promoted GSK3β accumulation in cardiac hypertrophy mice. Previous study has found that GSK-3β activation was involved in the process of cardiac hypertrophy (Chen et al., 2018). For example, previous research has found that overexpressed cardiac-specific Traf2 enhances cardiac hypertrophy by activating AKT/GSK3β signaling pathway (Huang et al., 2014). In addition, cardiac-specific mindin overexpression attenuates cardiac hypertrophy by blocking AKT/GSK3β and TGF-β1-Smad signaling (Yan et al., 2011). However, in our study, we found that the mRNA expression level of GSK-3β was down-regulated in PE-induced cardiomyocytes, furthermore, inhibiting SLC26A4 increased its expression in cardiomyocytes, which was opposed to previous studies. The expression of GSK-3β has been found to be mediated by many factors. Our results indicated that high SLC26A4 expression in PE-induced cardiomyocytes could inhibit the expression of GSK-3β.

Adaptation of protein turnover is a key process of cardiac hypertrophy, which is the balance between protein synthesis and degradation (Ullrich et al., 2019). The main mechanism controlling protein degradation is the ubiquitin-proteasome system, which is important for many cellular processes, including apoptosis (Xie et al., 2018). We showed that siRNA-SLC26A4 treatment could promote apoptosis of cardiomyocytes induced by PE, indicating that inhibiting SLC26A4 could remodel cardiac structure after cardiac hypertrophy through the induction of apoptosis.

Autophagy is an important regulator of cardiac hypertrophy (Qi et al., 2019). Dysregulation of autophagy has been demonstrated in cardiac hypertrophy, suggesting that targeting autophagy is a potential strategy for the treatment of cardiac hypertrophy. Cardiac-specific loss of autophagy-related genes promotes cardiac hypertrophy and heart failure induced by pressure overload (Huo et al., 2019). We detected the expression of autophagic markers including beclin-1, LC3II/I and P62 (Chen et al., 2016). Inhibiting SLC26A4 displayed enhanced autophagy indicated by lower beclin-1 and LC3II/I and higher P62 in PE-induced cardiac hypertrophy. LC3II/I and beclin-1 are recognized as autophagosome markers. Increasing evidence demonstrates that P62 is closely associated with autophagic process. P62 binds to ubiquitinated proteins via ubiquitin-associated domain and delivers them to autophagosomes for degradation (Qi et al., 2019). Therefore, our results showed that inhibiting SLC26A4 could suppress cardiac hypertrophy by autophagy inactivation of myocardial cells.

Reversing hypertrophy is the main treatment strategy for patients with cardiac hypertrophy. However, the effect of antihypertensive treatment on cardiac hypertrophy is not satisfactory (Oyabu et al., 2013). Therefore, the development of molecular targets for the treatment of patients with cardiac hypertrophy has become an effective treatment. Our findings revealed that SLC26A4 could become a potential therapeutic target for cardiac hypertrophy.

## Conclusion

Our study found that SLC26A4 is involved in cardiac hypertrophy and inhibiting SLC26A4 could decrease the release of ANP or BNP and promote the expression of GSK-3β *in vivo* and *in vitro*. In addition, inhibition of SLC26A4 could suppress autophagy and induce apoptosis of cardiomyocytes. Therefore, SLC26A4 could become a potential target for the treatment of cardiac hypertrophy, which provides new insights into the mechanisms and treatment of cardiac hypertrophy.

##  Supplemental Information

10.7717/peerj.8253/supp-1 Table S1 Immunofluorescence confocal detection assay-1

10.7717/peerj.8253/supp-2 Table S2 Q-PCR

10.7717/peerj.8253/supp-3 Table S3 Immunofluorescence confocal detection assay-3

10.7717/peerj.8253/supp-4 Table S4 Western blot

10.7717/peerj.8253/supp-5 Table S5 Apoptosis detection experiment

10.7717/peerj.8253/supp-6 Table S6 Western Blot detection gray value analysis

10.7717/peerj.8253/supp-7 Table S7 Optical density analysis

10.7717/peerj.8253/supp-8 Table S8 Body weight

10.7717/peerj.8253/supp-9 Table S9 Gels/Blots

## Abbreviations

SLC26A4: solute carrier family 26 member 4
PE: phenylephrine
LC3: light chain 3
ANP: atrial natriuretic peptide
BNP: brain natriuretic peptide
HE: haematoxylin–eosin
RT-qPCR: real-time quantitative polymerase chain reaction
α-SMA: α smooth muscle actin
GAPDH: Glyceraldehyde-3-phosphate dehydrogenase
AAC: abdominal aortic constriction
GFP: microtubule-associated protein
H&E: Haematoxylin–Eosin
Atgs: autophagy related genes

## Additional Information and Declarations

### Data Availability

The following information was supplied regarding data availability: The raw measurements are available in the Supplemental Files.
