# Supplementary figures and images for: Inhibiting SLC26A4 reverses cardiac hypertrophy in H9C2 cells and in rats
Source: PeerJ. 2020 Jan 21;8:e8253. doi: 10.7717/peerj.8253 (PMC6979409; doi:10.7717/peerj.8253)

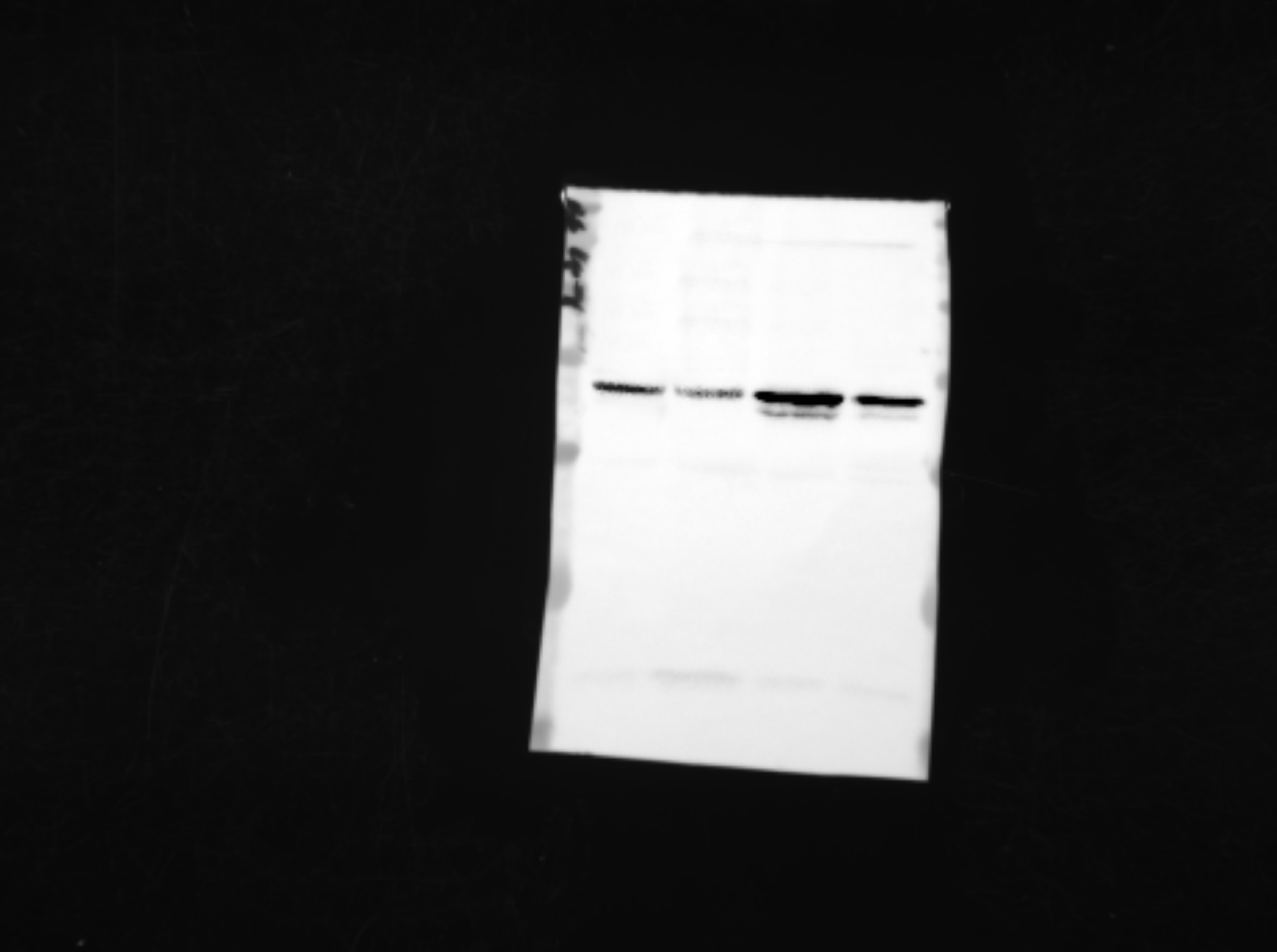

Supplement: Table S9 [file peerj-08-8253-s009.zip › Supplementary data 9/a-SMA.tif]

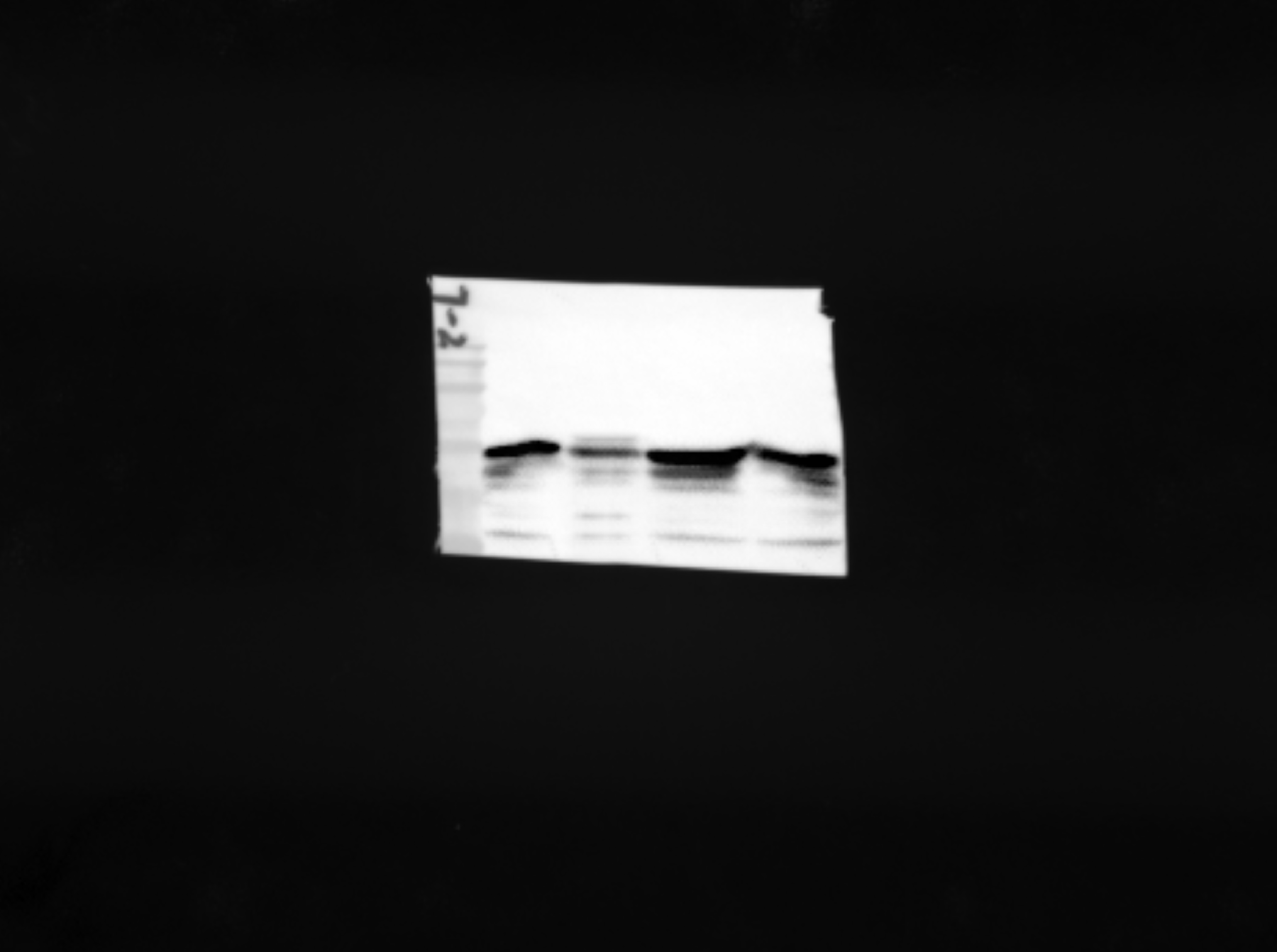

Supplement: Table S9 [file peerj-08-8253-s009.zip › Supplementary data 9/beclin-1.tif]

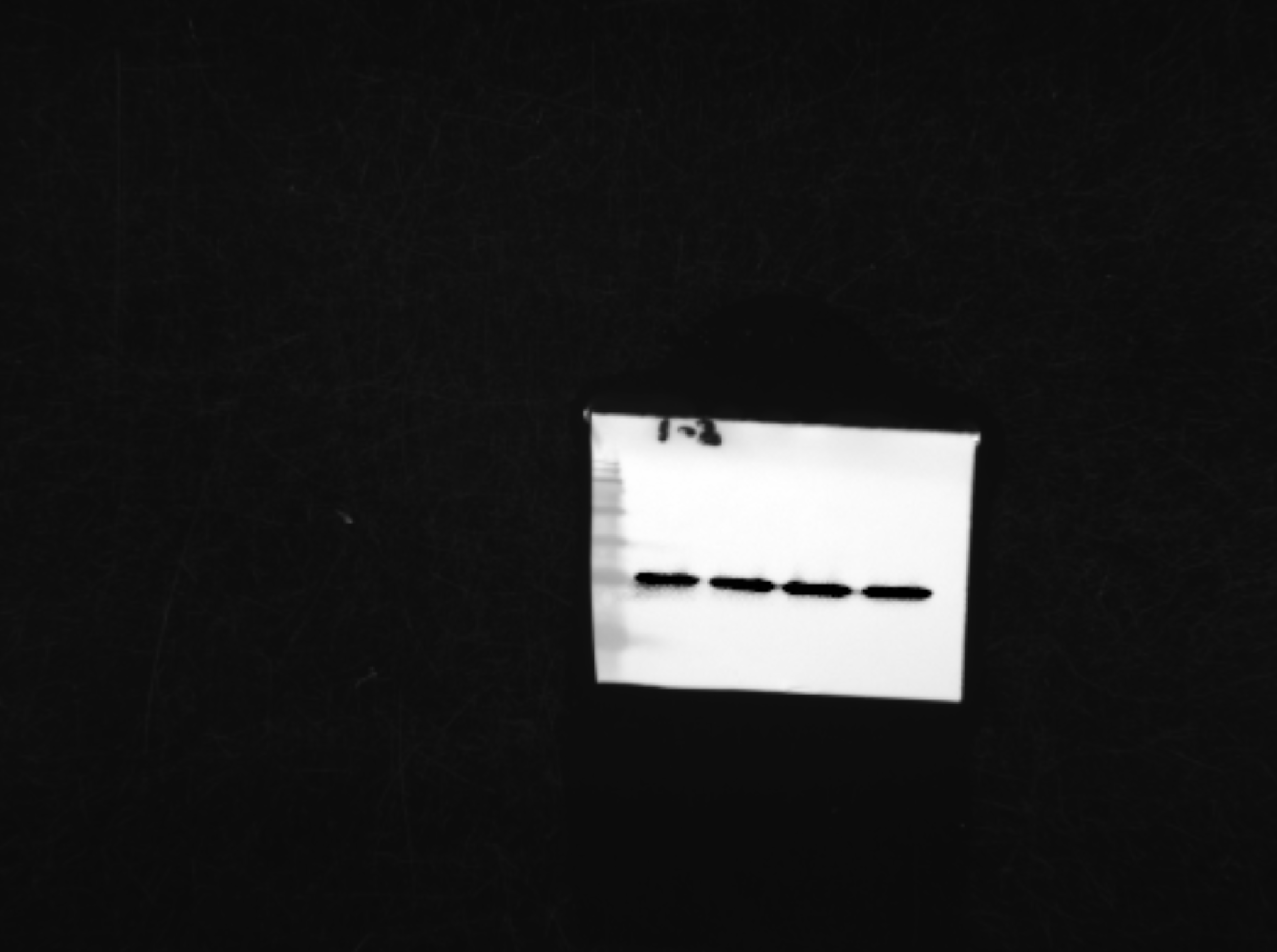

Supplement: Table S9 [file peerj-08-8253-s009.zip › Supplementary data 9/GAPDH.tif]

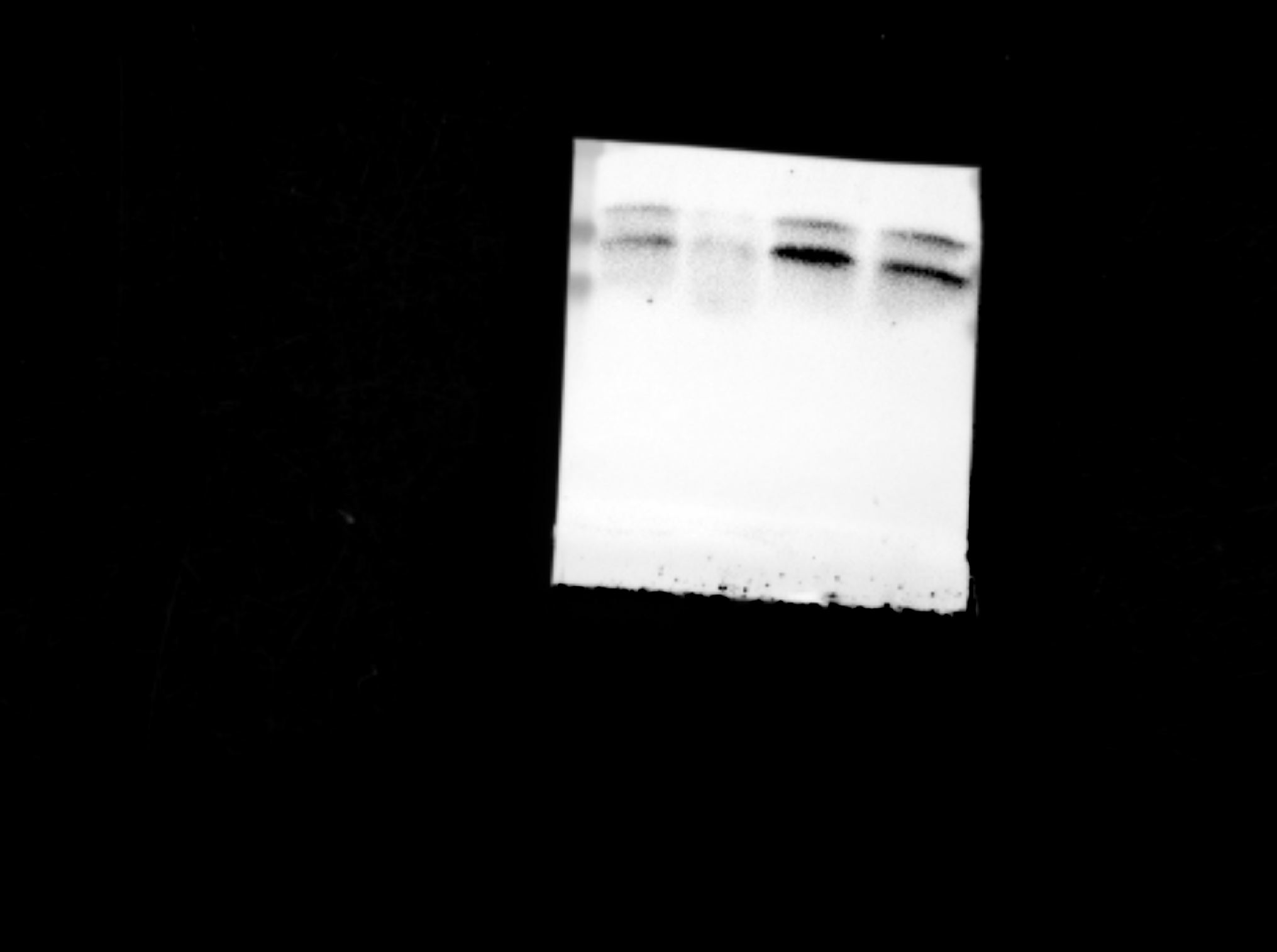

Supplement: Table S9 [file peerj-08-8253-s009.zip › Supplementary data 9/LC3.tif]

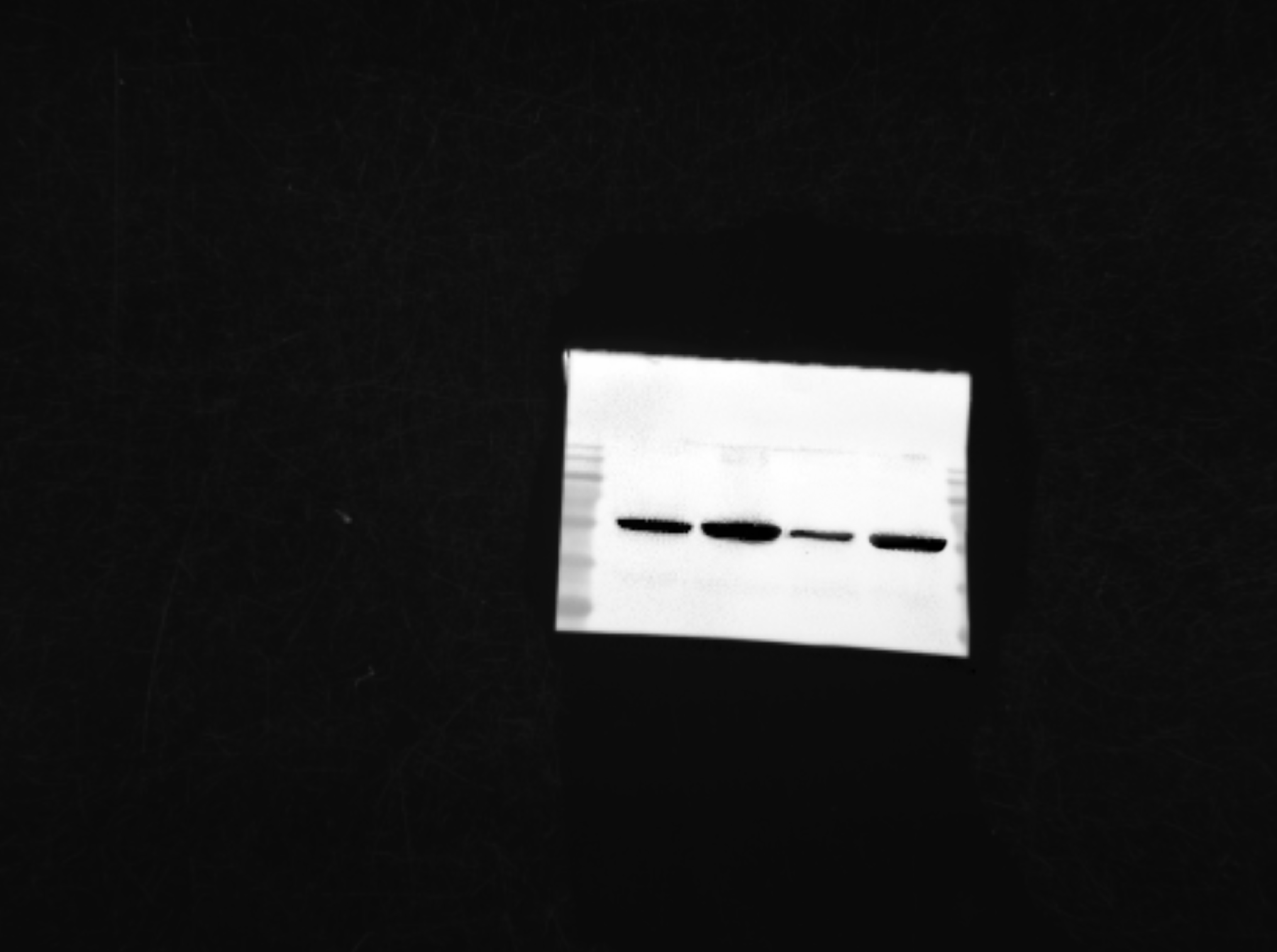

Supplement: Table S9 [file peerj-08-8253-s009.zip › Supplementary data 9/P62.tif]
